# Supplementary material for: Complexome profiling of the Chlamydomonas psb28 mutant reveals TEF5 as an early PSII assembly factor
Source: Plant Cell. 2025 Mar 17;37(6):koaf055. doi: 10.1093/plcell/koaf055 (PMC12164590; doi:10.1093/plcell/koaf055)
Supplement: koaf055_Supplementary_Data [file koaf055_supplementary_data.zip › psb28_interactive.zip!/plots/intensity/qualityPlots/peptides/_Cre12.g497850.t1.2.html]

### Cre12.g497850.t1.2

Trivial name:   
SeqCoverage [median]: 26.40  
Mass: 23.40 kDa  
  
 

| Sequence | MissedCleavage | Mass [Da] | wtIntensity | muIntensity | Score |
| --- | --- | --- | --- | --- | --- |
| ALENIR | 0 | 714.40 | 0.00 | 2440.43 | 77.75 |
| EMSPIQFDDDAGK | 0 | 1451.62 | 0.00 | 716.04 | 121.90 |
| GTFFAVR | 0 | 796.42 | 0.00 | 3800.61 | 80.56 |
| LDMEVPGAMLFR | 0 | 1377.68 | 375.82 | 4148.67 | 117.30 |
| NVVGLISMAEGAEAGQEAK | 0 | 1872.93 | 1500.76 | 5895.71 | 143.38 |
|  |  |  | 1876.6 | 17001.5 |  |
